# A Roadmap for Efficient and Stable All-Perovskite Tandem Solar Cells from a Chemistry Perspective

**DOI:** 10.1021/acscentsci.2c01077

**Published:** 2022-11-07

**Authors:** Pu Wu, Deepak Thrithamarassery Gangadharan, Makhsud I. Saidaminov, Hairen Tan

**Affiliations:** † National Laboratory of Solid State Microstructures, Jiangsu Key Laboratory of Artificial Functional Materials, College of Engineering and Applied Sciences, Frontiers Science Center for Critical Earth Material Cycling, Nanjing University, Nanjing210023, P. R. China; ‡ Department of Chemistry, University of Victoria, Victoria, British ColumbiaV8P 5C2, Canada

## Abstract

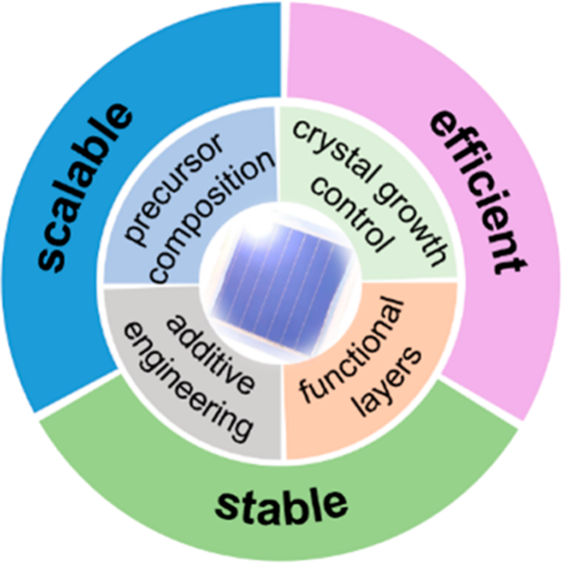

Multijunction tandem solar cells offer a promising route to surpass the efficiency limit of single-junction solar cells. All-perovskite tandem solar cells are particularly attractive due to their high power conversion efficiency, now reaching 28% despite being made with relatively easy fabrication methods. In this review, we summarize the progress in all-perovskite tandem solar cells. We then discuss the scientific and engineering challenges associated with both absorbers and functional layers and offer strategies for improving the efficiency and stability of all-perovskite tandem solar cells from the perspective of chemistry.

## Abstract 2

This Outlook presents the advances and challenges for all-perovskite tandem solar cells and provides insights into approaching highly efficient and stable all-perovskite tandem devices.

## 1 Introduction

In recent years, developing green energy solutions to meet increasing global energy demand in the wake of concerns over climate change has been at the forefront of chemical sciences.^1^ Photovoltaics, which directly converts sunlight to electricity, is considered to be a practical and sustainable solution because solar energy is abundant and eco-friendly.^2^ To allow this technology to be more competitive and accessible, manufacturing costs should be brought down. Increasing the power conversion efficiency (PCE) per unit area is key to further reducing the overall cost of photovoltaics. Multijunction tandem solar cells potentially can reach PCEs beyond 40%, which is superior to the 33% of single-junction devices, arousing enormous scientific and industrial interests around the world.^3,4^

**Figure 1:**
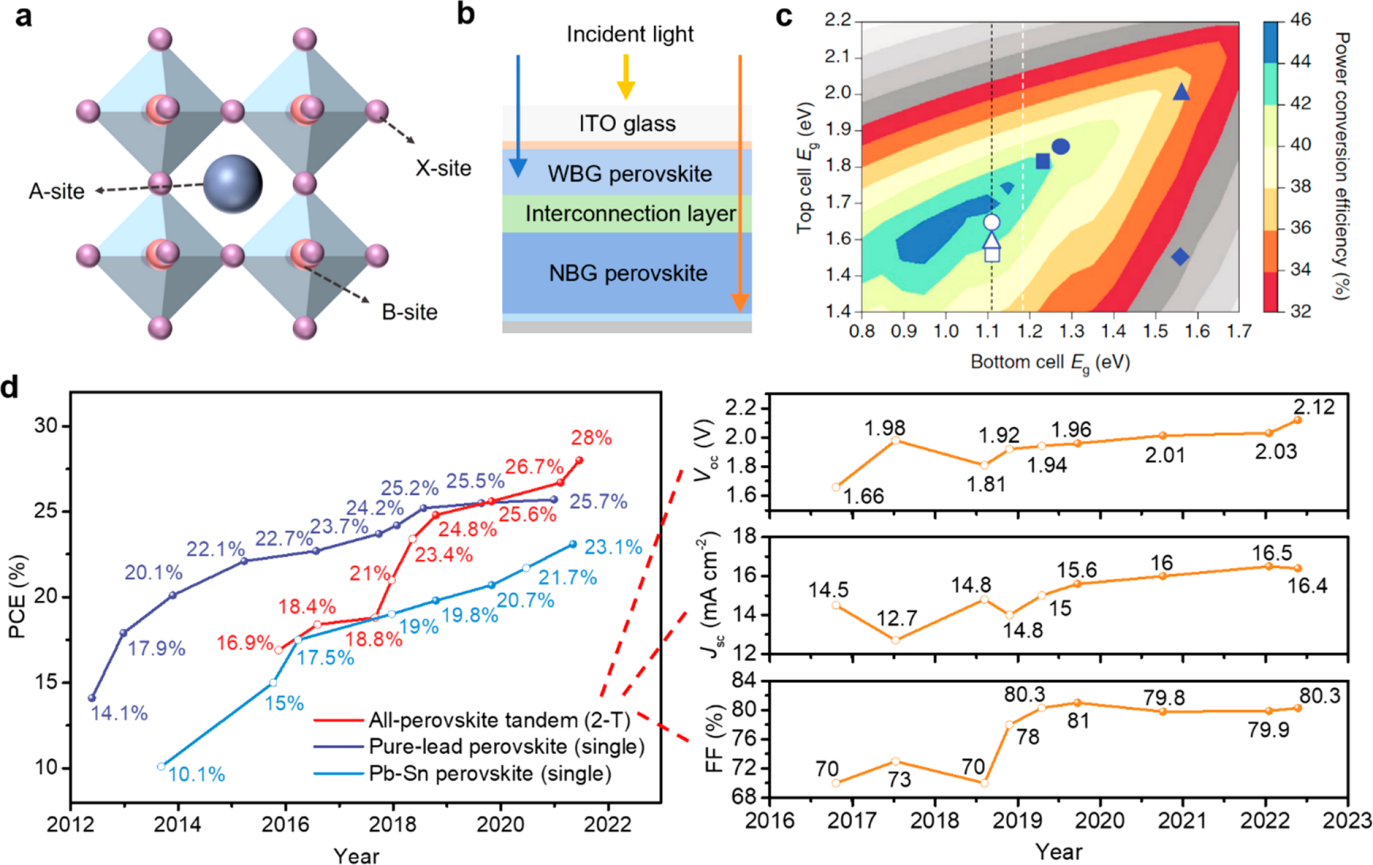
Basics and evolution of all-perovskite tandem solar cells. (a) Schematic representation of the crystal structure of an organic–inorganic metal halide perovskite. (b) Schematic illustration showing the structure of a monolithic all-perovskite tandem solar cell. (c) Theoretical efficiency limit for 2-T tandems with different bandgap of subcells. (d) PCE evolution and corresponding photovoltaic parameters of monolithic all-perovskite tandem solar cells (certified data is represented by a solid circle, and uncertified data is represented by a hollow circle). Panel (c) reproduced with permission from ref (3). Copyright 2018 Springer Nature.

Organic–inorganic metal halide perovskites are arguably the most promising candidates for next-generation solar energy absorbers owing to their impressive optical and electrical properties, e.g., direct bandgap transition, high absorption coefficient, low exciton bonding energy, and long charge-carrier diffusion length.^5^ The empirical formula of perovskite is ABX_3_, where A represents a monovalent cation like methylammonium (MA^+^), formamidinium (FA^+^), or cesium (Cs^+^); B represents a divalent metal cation like Pb^2+^ or Sn^2+^; and X represents a monovalent anion like halide anions (I^–^ or Br^–^) (Figure 1a). The bandgap of perovskites can be tuned through composition engineering, making them excellent candidates for multijunction solar cells.^6^ Moreover, the certified record PCE of single-junction perovskite solar cells has quickly reached 25.7% over merely a decade, which is competitive with traditional photovoltaics such as silicon, boosting the development of perovskite-based tandem solar cells.^7^

Among perovskite-based tandem solar cells, all-perovskite tandem solar cells can sufficiently leverage the unique advantages of perovskite materials, including high performance, low cost, easy fabrication process, and compatibility with flexible substrates. Monolithic all-perovskite tandems have reached 28% PCE, and a breakthrough >30% efficiency is expected.^8^ In this outlook, we will briefly review the development of all-perovskite tandem solar cells. Then we will discuss the relevant scientific and engineering challenges of perovskite subcells and provide some strategies for performance improvement through precursor composition, crystal growth control, and additive engineering. Subsequently, the progress and perspectives of functional layers concerning charge recombination and extraction in tandem devices are discussed. We attempt to draw a roadmap for designing high-performance and stable all-perovskite tandem solar cells.

## 2 Fundamentals and Evolution of Tandem Photovoltaic Devices

In a tandem device, semiconductors with different bandgaps are stacked together to expand the utilization of the solar spectrum and mitigate the thermalization losses.^9^ On the basis of the connection of subcells, a double-junction tandem device generally falls into two configurations: two-terminal (2-T) and four-terminal (4-T). The 2-T (or monolithic) tandem device is fabricated with two subcells connected by an interconnection layer (ICL), whereas the 4-T tandem device is realized by mechanically stacking two separate cells together. Compared with a 4-T configuration, a 2-T tandem design can minimize the spectral losses of electrodes and avoid additional manufacturing costs, making it more attractive for practical applications. Thus, recent research has been focused on developing efficient and stable monolithic tandem solar cells.

As shown in Figure 1b, a monolithic tandem solar cell is typically designed with a front wide bandgap (WBG) subcell collecting most of the high-energy photons and a back narrow bandgap (NBG) subcell absorbing the remaining low-energy photons. The theoretical calculations show that the PCE of the tandem solar cell is primarily dictated by the bandgap of the subcells, and a maximum PCE of ∼44% can be achieved by pairing a ∼1.8 eV WBG perovskite with a ∼1.2 eV NBG perovskite (Figure 1c).

The surge in the development of single-junction perovskite solar cells has promoted the efficiency enhancement of perovskite-based tandem devices. Figure 1d displays the evolution of record efficiencies of pure-Pb, Pb–Sn single-junction perovskites, and monolithic all-perovskite tandem solar cells. Meanwhile, the evolution in photovoltaic parameters of the tandem cells is exhibited separately for a better understanding of how to improve the PCE, where *V*_oc_, *J*_sc_, and FF are open-circuit voltage, short-circuit current density, and fill factor, respectively. Since 2019, the *V*_oc_ and *J*_sc_ have been gradually increasing, whereas the FF value is hovering around 80%. According to the empirical estimation, these parameters are likely to increase further, and the *V*_oc_, *J*_sc_, and FF are expected to reach nearly 2.28 V, 18 mA cm^–2^, and 82%, respectively.^10^ Thus, to reach 33.6% PCE for all-perovskite tandem solar cells, an insightful understanding of perovskite absorbers and functional layers is needed.

## 3 Optimization of Perovskite Absorbers

For all-perovskite tandem solar cells, it is still challenging to reduce the voltage losses (defined as *E*_g_ – *qV*_oc_) of the WBG perovskite and obtain NBG perovskites with high efficiency and good stability. This section will discuss strategies to mitigate these issues through precursor composition, crystal growth control, and additive engineering.

### 3.1 Wide-Bandgap Perovskite

#### 3.1.1 Bandgap Tunability and Suppression of Phase Segregation

The common strategy to enlarge the bandgap of perovskites is to mix Br with I at the X-site. For example, the bandgap of the MAPbX_3_ perovskite can be continuously tuned from 1.58 to 2.28 eV as the Br/I ratio increases from 0% to 100%.^11^ However, MAPb(I_1–*x*_Br_*x*_)_3_ perovskite materials with a high ratio of Br/I (>20%) tend to phase segregate into lower-bandgap I-rich minority and higher-bandgap Br-rich majority domains, which is known as the Hoke effect.^12^ The Hoke effect results in a redshift of photoluminescence (PL) and an increase in electronic disorder, limiting the achievable *V*_oc_ and photostability of these absorbers.

**Figure 2:**
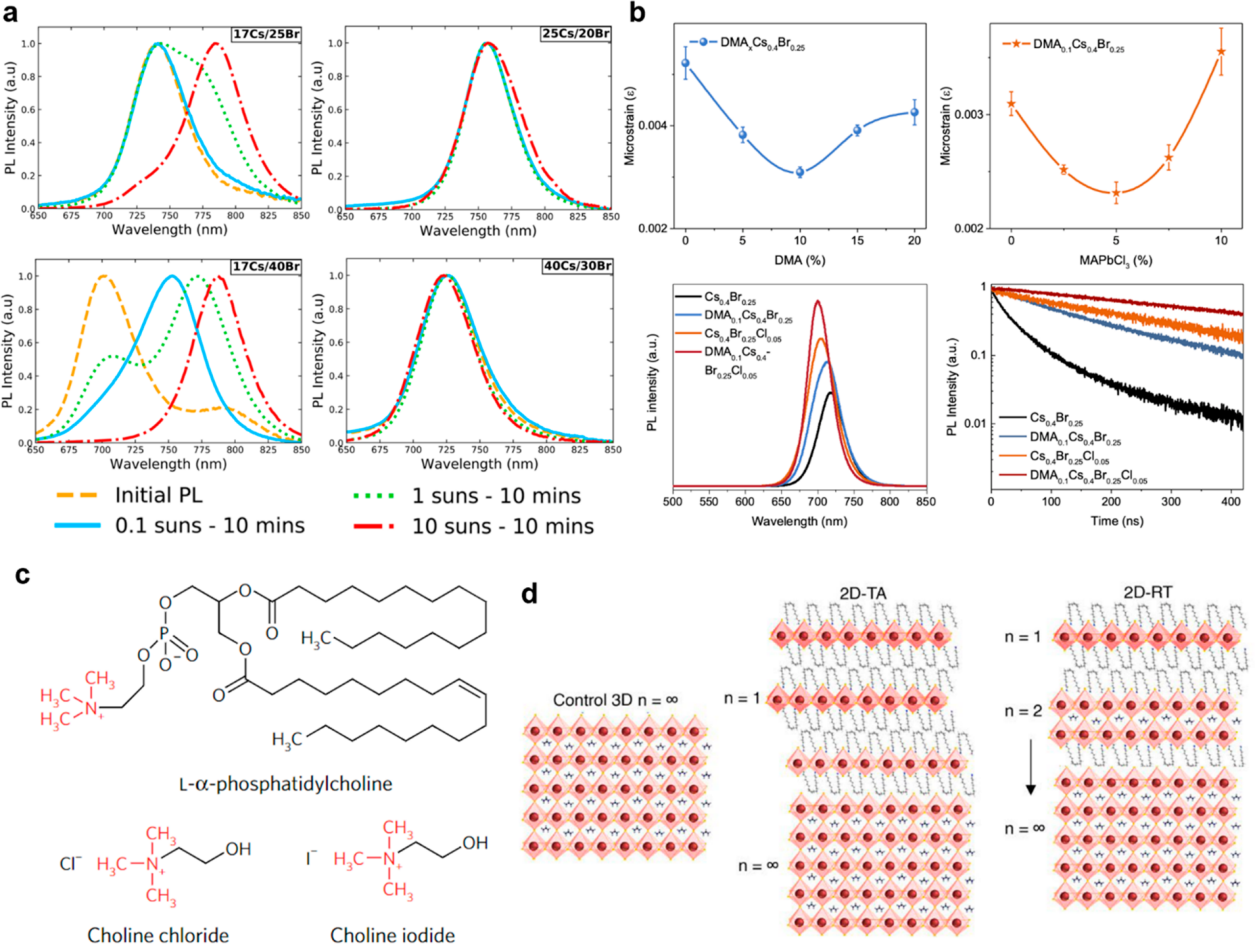
Optimization of wide-bandgap absorbers for all-perovskite tandem solar cells. (a) Comparison PL data of 1.68 eV bandgap perovskites (Cs17/Br25 or Cs25/Br20) and ∼1.75 eV bandgap perovskites (Cs17/Br40 and Cs40/Br30). Reproduced with permission from ref (15). Copyright 2018 American Chemical Society. (b) Lattice strain analysis and charge carrier dynamics of WBG perovskite films. Reproduced with permission from ref (23). Copyright 2022 Wiley. (c) Chemical structures of l-α-phosphatidylcholine, choline iodide, and choline chloride. Reproduced with permission from ref (26). Copyright 2017 Springer Nature. (d) Schematic illustration of 2D perovskite passivation with different *n* layers under thermal annealing at 100 °C (TA) and room-temperature process (RT). Reproduced with permission from ref (29). Copyright 2022 American Association for the Advancement of Science.

Composition engineering offers a straightforward approach to suppress photoinduced phase segregation and improve the performance of the WBG perovskite while it remains the desired bandgap. It appears that the composition of A-site cations has a strong influence on the halide segregation behavior. Snaith and co-workers found that FA_0.83_Cs_0.17_Pb(Br_0.4_I_0.6_)_3_ exhibited restrained phase segregation compared with MAPb(I_0.6_Br_0.4_)_3_ perovskite, as well as much decreased energetic disorder.^13^ Herz and co-workers demonstrated that, for the MA mixed-halide perovskite, the low-barrier ionic pathways resulted in facile halide rearrangement in minority regions of the perovskite absorber, whereas the FACs counterpart lacked such ionic pathways and exhibited, consequently, restrained propensity to halide segregation.^14^ Moreover, it was reported by McGehee and co-workers that using relatively high Cs content at the A-site rather than Br at the X-site is preferable in realizing optimal bandgap with improved PCE and photostability simultaneously.^15^ They demonstrated that perovskites containing 25Cs/20Br or 40Cs/30Br compositions showed better photostability than the compositions of high Br content with the same bandgap (Figure 2a).

Incorporation of an appropriate amount of larger A-site cations, such as guanidinium (Gua), dimethylammonium (DMA), and acetamidinium (AC), into the lattice also widens the bandgap of perovskites via lattice strain.^16−18^ For example, Moore and co-workers designed a photostable WBG perovskite with a bandgap of 1.7 eV containing only 20% Br through cation tuning.^16^ Sargent and co-workers developed a Br-free WBG perovskite by incorporating DMA or Gua into the CsPbI_3_ perovskite.^19^ Interestingly, Kanatzidis and co-workers reported a new bandgap-tuning mechanism of perovskite materials through the inclusion of small molecules such as ethylenediammonium (en).^20,21^ The use of en produces a highly disrupted, hollow perovskite structure, resulting in an enlarged bandgap because of the elimination of several B-X connections. The bandgap of MA_1–*x*_(en)_*x*_Pb_1–0.7*x*_I_3–0.4*x*_ perovskites can be achieved from 1.53 to 2.1 eV when the *x* value varies from 0.03 to 0.44, offering the potential for its utilization in tandem solar cells.

In addition, a triple-halide strategy, partially alloying Br with Cl, could also suppress the halide segregation.^22^ Moreover, our recent study showed that alloying both at the A-site and X-site can modulate the crystal structure and electron orbital configuration synchronously, which allowed for obtaining ∼1.8 eV WBG perovskites with only 25% Br.^23^ Through systematic compositional engineering, we found that adding both DMAI and MAPbCl_3_ (10 mol % of DMAI and 5 mol % of MAPbCl_3_) into the Cs_0.4_FA_0.6_Pb(I_0.75_Br_0.25_)_3_ perovskite enabled the optimal bandgap for tandem and minimized the lattice strain and trap densities simultaneously (Figure 2b). Thus, the improved WBG perovskite, with a high PCE of 17.7% and a *V*_oc_ of 1.26 V, exhibited considerably suppressed light-induced phase segregation and maintained 90% of their initial performance after 1 000 h maximum power point (MPP) tracking under 1 sun illumination.

The concept of manipulating the octahedral tilt by incorporating cations in WBG perovskites to alter the bandgap with no use of Br in the composition is a promising approach. So far, the addition of DMA into perovskite compositions has been found to be a very efficient strategy for widening the bandgap without inflicting phase instability. We believe that there is plenty of room for bandgap tunability without inducing phase segregation by using other cations with different sizes through steric engineering.

#### 3.1.2 Bulk or Interface Passivation for Reduced *V*_oc_ Losses

The WBG perovskite subcell provides most of the *V*_oc_ in a tandem device; therefore, reducing the voltage loss of the WBG perovskite is crucial to reaching high PCEs in tandem solar cells. Previously, the large voltage loss was ascribed to photoinduced phase segregation in mixed halide perovskites. However, recent reports indicated that the loss mainly stems from the relatively low radiative efficiency of the bulk absorber and severe nonradiative recombination at the interface between the perovskite and the charge-transport layers, rather than from halide segregation.^24,25^ Therefore, it is important to modify the bulk and surface of perovskites for higher *V*_oc_ and hence better performance.

Post-treatment is an effective method of decreasing the defect densities at the surface of WBG perovskite films and thus improving the performance and stability. Huang and co-workers used quaternary ammonium halides (Figure 2c), which consisted of a zwitterion structure, to passivate both negatively (such as I^–^ vacancies) and positively (such as MA^+^ vacancies) charged defects for pure-lead perovskites with different bandgaps, leading to significant *V*_oc_ enhancement on all types of devices.^26^ In addition, employing alkylammonium organic cations, such as phenylethylammonium (PEA),^27^*n*-butylammonium (BA),^28^ and oleylammonium (OA),^29^ to establish 2D-perovskite passivation layers provides another route to enhance the *V*_oc_ as well as the stability of WBG perovskites. The dimensionality (or *n* value) of the 2D-perovskite fragment is critical to the surface passivation and carrier transport dynamic. De Wolf and co-workers reported that the higher-dimensionality 2D-perovskite layer (*n* ≥ 2) tended to form at room temperature (2D-RT) with the OAI molecule, whereas the *n* = 1 layers appeared after thermal annealing treatment (2D-TA) (Figure 2d).^29^ The surface of perovskites treated with OAI at room temperature exhibited enhanced n-type character, resulting in more efficient electron-selective transfer between the perovskite and C_60_. This enabled significant improvement in the voltage and intrinsic stability of devices.

On the other hand, the additives added to the bulk materials, e.g., 1-butyl-1-methylpiperidinium tetrafluoroborate, can strongly influence not only the grain boundaries of perovskites but also the interfaces between the perovskite and charge-transport layers, probably because these additives help to form a preferable interface between the perovskite surface with reduced trap density.^30^ Thus, a combination of bulk and interface passivation could be promising for reducing defect density, suppressing trap-assisted recombination, and improving WBG perovskite performance. Incorporating a 2D perovskite phase in the form of 2D/3D heterostructures in bulk, interfaces, and grain boundaries has been proven to reduce *V*_oc_ loss in WBG perovskites. 2D perovskites containing bifunctional organoammonium cations, such as Y(CH_2_)_2_NH_3+_ (Y = F, Cl, Br, I, CN) are still not widely explored for designing 2D/3D heterostructures.

### 3.2 Narrow-Bandgap Perovskites

Partial substitution of Pb with Sn in perovskite composition is the most effective approach to obtaining NBG perovskites for high-performance all-perovskite tandem devices. However, tin-based perovskite solar cells usually show inferior performance and stability to those of only lead perovskite cells, primarily because of (i) poor film morphology due to fast and uncontrolled crystallization, i.e., pinholes, heterogeneous nucleation, and rough surface,^31,32^ and (ii) facile oxidation of Sn^2+^ to Sn^4+^ inducing severe p-type self-doping, and consequently this results in severe nonradiative recombination.^33^

#### 3.2.1 Crystallization Modulation for High-Quality Films

**Figure 3:**
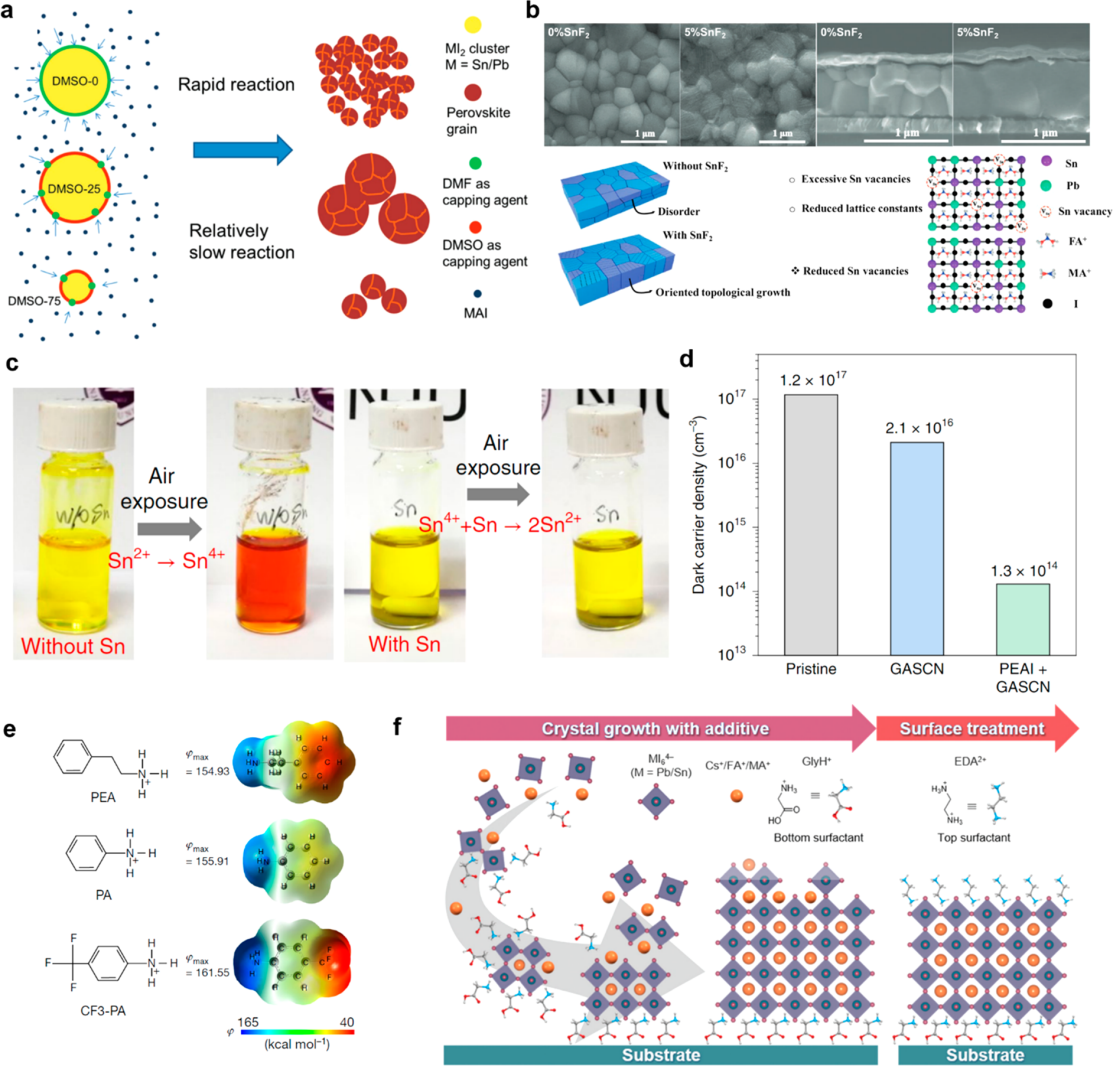
Optimization of narrow-bandgap absorber layers for all-perovskite tandem solar cells. (a) Schematic illustration of the reaction mechanism for Pb–Sn perovskites with different DMSO/dimethylformamide (DMF) solvent ratios. (b) Scanning electron microscopy (SEM) images and schematic diagram of (FASnI_3_)_0.6_(MAPbI_3_)_0.4_ films under the influence of SnF_2_. (c) Photographs of Pb–Sn perovskite precursor solutions without and with metallic Sn powders, taken before and after air exposure. (d) Dark carrier density of Pb–Sn PSCs prepared with and without additives. (e) Molecular structures and corresponding Gaussian calculated electrostatic potentials (φ) of three passivators (PEA, PA, and CF3-PA). (f) Schematic illustration of a Pb–Sn perovskite film treated with the GlyHCl additive and EDAI_2_ surface modification. Panels (a) and (b) reproduced with permission from refs (34 and 35), respectively. Copyright 2017 and 2021 Wiley, respectively. Panels (c–e) reproduced with permission from refs (50, 62, and 64), respectively. Copyright 2019, 2022, and 2022 Springer Nature, respectively. Panel (f) reproduced with permission from ref (65). Copyright 2022 Royal Society of Chemistry.

Optimizing the chemistry of a precursor solution is an effective way to control the nucleation and crystal growth of perovskites. For instance, Choy and co-workers introduced dimethyl sulfoxide (DMSO) to control the crystallization rate of the MASn_0.25_Pb_0.75_I_3_ perovskite and achieved a high-quality perovskite film with smooth surfaces, preferential orientation, and high crystallinity. They demonstrated that an optimized amount of DMSO in precursor solution increased the crystal size of Sn–Pb perovskites and ameliorated inhomogeneous Sn/Pb distributions (Figure 3a).^34^ Additionally, Chen et al. found that the widely used tin fluoride (SnF_2_) not only suppresses the oxidation of Sn^2+^ but also facilitates the topological growth of Sn–Pb perovskite grains, leading to a solar cell performance of 20.27% (Figure 3b).^35^

Among the various methods to fabricate perovskite films, solvent engineering is the most popular method for highly efficient tin-based perovskites. Several studies have lent insight into the optimal application of antisolvent. Liao et al. compared the morphology of FASnI_3_ perovskite films deposited on poly(3,4-ethylenedioxythiophene):polystyrene sulfonate (PEDOT:PSS) by different antisolvent drippings (chlorobenzene, toluene, and diethyl ether).^36^ They found that diethyl ether produces highly uniform and compact films with full coverage, whereas chlorobenzene and toluene lead to rough and hierarchical morphology with pinholes. On the other hand, Bandara et al. showed that the use of toluene as an antisolvent enables larger grains of the Pb–Sn perovskite and, more importantly, aids in the removal of the Sn^4+^ defects in the absorber, resulting in enhanced device performance.^37^ Interestingly, Cao et al. reported that the vertical Pb/Sn compositional gradient and the microstructure of the Pb–Sn perovskite varied with the antisolvent temperature.^38^ The antisolvent with a lower temperature lowered the Pb/Sn ratio on the surface of the FA_0.5_MA_0.45_EA_0.05_Sn_0.5_Pb_0.5_I_3_ film and increased the perovskite compositional gradient across the bulk, which was preferable for photogenerated carrier separation and collection. They also found that colder antisolvent slows down the nucleation rate and leads to larger grain sizes. The optimized device yields a high PCE up to 22.02% with a *V*_oc_ of 0.88 V. To date, there are only a few studies on the nucleation and growth process of Pb–Sn mixed perovskites, while it has a significant effect on device performance and stability.^34,38,39^ Gas quenching during perovskite film deposition has also been found to be beneficial to achieve high-quality perovskite films. It has been reported that the MA-free Sn/Pb perovskites deposited through the gas quenching method led to a smoother surface than the antisolvent-treated films.^40^

In addition, controlling the annealing process is essential to regulating the crystallization dynamics for perovskite films. Recently, Li and co-workers developed a close-space annealing (CSA) strategy, which involves covering the intermediate-phase perovskite with a solvent-permeable film during the annealing process.^32^ In comparison with the normal annealing, the CSA method slowed the vertical solvent-escape rate and led to recrystallization of grains and a smooth surface. This approach enabled the fabrication of a Pb–Sn perovskite solar cell (PSC) with a 21.5% PCE. Thus, the crystallization property is affected by not only the precursor solution and antisolvent but also the annealing process, and all these factors need to be controlled precisely during the NBG perovskite-fabrication process.

#### 3.2.2 Oxidation and Stability of NBG Materials

The stability of NBG perovskites, especially Sn-based materials, is another major concern in realizing highly efficient and stable tandems. Although tin has a similar valence electron configuration (ns^2^np^2^) to that of lead, Sn tends to lose all its valence electrons easier than Pb due to the lack of lanthanide shrinkage.^41^ Thermogravimetric and ultraviolet–visible spectrophotometry analyses reveal that pure Sn perovskite and Pb–Sn perovskite follow different oxidation mechanisms, as summarized in eqs 1 and 2.^42^
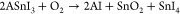
1
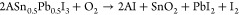
2Alloying Sn with Pb reduces the probability of having multiple adjacent tin atoms in the perovskite structure, forcing the oxidation reaction toward a less favorable way where more Sn–I and Pb–I bonds are required to break to form I_2_. Unfortunately, it remains ambiguous whether and how tin-based perovskites are degraded without the presence of oxygen or vapor. A comprehensive understanding of the oxidation and degradation mechanism of tin-containing perovskites is necessary to further improve the stability of all-perovskite tandem solar cells.

Tin-containing perovskites are prone to ready oxidation of Sn^2+^ to Sn^4+^ in the precursor and during film formation, which leads to tin vacancies and hole doping in the perovskites, affecting their performance and stability. Addition of SnF_2_ in the composition is most effective and widely used to suppress Sn^2+^ oxidation and, thereby, the hole doping.^36,43,44^ The role of SnF_2_ in mitigating the oxidation of Sn^2+^ is largely studied in single A cation, pure-Sn perovskite compositions; there is no consensus on how they influence the properties of multication, Pb–Sn perovskite compositions, which are widely used in tandem applications. Herz and co-workers argued that the addition of 1% SnF_2_ significantly reduces the background hole density in the Sn–Pb perovskite, contrary to the commonly used 20% SnF_2_ in pure-Sn perovskites.^45^ The exact mechanism of prevention of the oxidation of Sn^2+^ is still not clear; two prominent hypotheses are (1) the excess Sn from SnF_2_ reduces Sn vacancies by making their formation less plausible and (2) SnF_2_ lowers the rate of oxidation.

Adding reducing agents and antioxidants, such as hypophosphorous acid,^46,47^ ascorbic acid (AA),^48^ formamidine sulfinic acid,^49^ etc., is an effective way to hinder the oxidation of Sn^2+^ in the perovskite. We have found that the introduction of metallic Sn powders into Sn–Pb perovskite precursor ink suppresses the oxidation of Sn^2+^ to Sn^4+^ through a comproportionation reaction (Sn^4+^ + Sn^0^ → 2Sn^2+^), resulting in improved performance and stability (Figure 3c).^50^ Recently, Graham and co-workers investigated the redox behavior of these additives and summarized their functional mechanisms into three kinds: (1) through halide exchange to debilitate the harmful impact of Sn^4+^; (2) through redox behavior to reduce Sn^4+^ to Sn^2+^; and (3) through coordination with Sn^2+^ at the interface or reacted with oxygen as a sacrificial antioxidant to prevent tin oxidation.^51^ Thus, while selecting or designing effective additives for NBG perovskites, their ability for halide exchange, the reduction potential, and the ability to bond with Sn species should be considered.

Cation composition also has a significant impact on the intrinsic stability of Pb–Sn perovskites. It has been demonstrated that incorporating an appropriate amount of Cs into MA- or FA-based Pb–Sn perovskites effectively modulates the crystallization process of films, leading to enhanced efficiency and stability.^52^ Further, reducing the content of MA by substituting the MA cation with FA and Cs cations has been reported to provide more thermally and operationally stable perovskites.^31,53,54^ Through temperature-programmed desorption mass spectrometry, Yan and co-workers found that the (FASnI_3_)_0.6_(MAPbI_3_)_0.4_ film decomposes at ∼68 °C, whereas the FA_0.85_MA_0.1_Cs_0.05_Sn_0.5_Pb_0.5_I_3_ releases an organic gas at ∼125 °C, confirming that reducing the proportion of MA is beneficial for improving the intrinsic stability of the NBG perovskites.^31^ However, the efficiencies of MA-free Pb–Sn perovskite solar cells still lag behind those of their MA-containing counterparts; further studies on their crystallization dynamics are needed to improve the quality of the films and, therefore, the performance of these stable perovskite devices.^55−58^

#### 3.2.3 Additive Engineering and Defect Passivation

Additives, such as halide or pseudohalide anions, metal cations, and ammonium cations, can reduce energy disorder and enhance carrier diffusion length via defect passivation in Sn-based perovskites.^59,60^ Zhu and co-workers found that the addition of 7 mol % GuaSCN into a Pb–Sn perovskite reduces the defect densities to >1 order of magnitude and increases the carrier diffusion length to 2.5 μs, which led to an exceptional PCE of 20.5% for a 1.25 eV bandgap perovskite.^61^ Recently, the same group reported that mixing an appropriate proportion of PEAI and GuaSCN further improves the optoelectronic properties of Sn–Pb perovskites through the formation of quasi-2D structure (PEA)_2_GuaPb_2_I_7_.^62^ The addition of this quasi-2D structure reduces the dark carrier density and enhances the bulk carrier lifetime, boosting the PCE of the NBG perovskite solar cells over 22% (Figure 3d). Sargent and co-workers found that PEAI-dissolved antisolvent can passivate defects both at the surface and within the perovskite films yet avoid the excess formation of the 2D phase perovskites to block the charge carrier transport.^63^ This resulted in a Pb–Sn perovskite with a certified PCE of 18.95% and greatly improved operational stability. In one of our studies, we presented that the −NH_3_^+^ side of 4-trifluoromethyl-phenylammonium (CF3-PA) had higher electrostatic potential than PEA (Figure 3e), which exhibited a stronger surface-passivator interaction with the perovskite.^64^ When adding a small amount of CF3-PA in the Pb–Sn perovskite precursor ink, we increased the carrier diffusion length of perovskites to >5 μm and achieved a high PCE of 22.2% for the NBG perovskite with an absorber thickness of ∼1.2 μm. This enabled a certified PCE of 26.4% in all-perovskite tandem solar cells.

Also, treating the perovskite surface with functional compounds passivates the defects. Ethylenediamine (EDA), a well-known electron donor, can bond to the positively charged defects or undercoordinated Sn^2+^ bonds on the perovskite surface.^65,66^ Hayase and co-workers revealed that post-treatment of the Pb–Sn perovskite with EDA reduces the density of structural defects and even transforms the perovskite surface from p-type nature to n-type, helping to form a graded band structure to facilitate the charge transport.^67^ Wakamiya and co-workers took this a step further and employed a comodifier of glycine hydrochloride (GlyHCl) additive and ethylenediammonium diiodide (EDAI_2_) post-treatment into Pb–Sn PSCs (Figure 3f). The GlyH^+^ cation, which tended to aggregate near the bottom interface of the perovskite, led to improved film crystallinity and facilitated hole extraction. This advance enabled Pb–Sn PSCs with a *V*_oc_ of 0.88 V and a PCE up to 23.6%.^65^ Recently, Ning and co-workers used 2-thiopheneethylamine thiocyanate (TEASCN) for surface treatment of Pb–Sn perovskite films to form a bilayer quasi-2D structure on the perovskite surface, which could passivate defects and ensure effective carrier transport simultaneously, resulting in improved device performance.^68^

As we discussed, oxidation of Sn^2+^ and subsequent formation of Sn^4+^ and Sn vacancies is a critical issue concerning Sn-based NBG perovskites devices. The oxidation could transpire in the precursor solution, during fabrication of the perovskite layer, or/and during the subsequent deposition of charge-transport layers and electrode on the top of the perovskite layer. Various additives in the precursor solution have been found effective in preventing oxidation of Sn during fabrication. To protect the perovskite layer from oxidation during the processing of other layers, an antioxidant capping layer can be deposited. There are only very few reports regarding antioxidant capping layers to safeguard Sn-based perovskite layers from oxidation, and more attention should be given to developing robust antioxidant capping layers. This strategy becomes more important and indispensable in tandem manufacturing at an industrial scale.

## 4 Optimization of Functional Layers

### 4.1 Interconnection Layer

In monolithic tandem devices, the ICL plays an essential role in electrically interconnecting each subcell. An ideal ICL should provide excellent ohmic contact with effective carrier recombination, good compatibility with fabrication processes, and high optical transparency, which enables minimal voltage and current losses.

**Figure 4:**
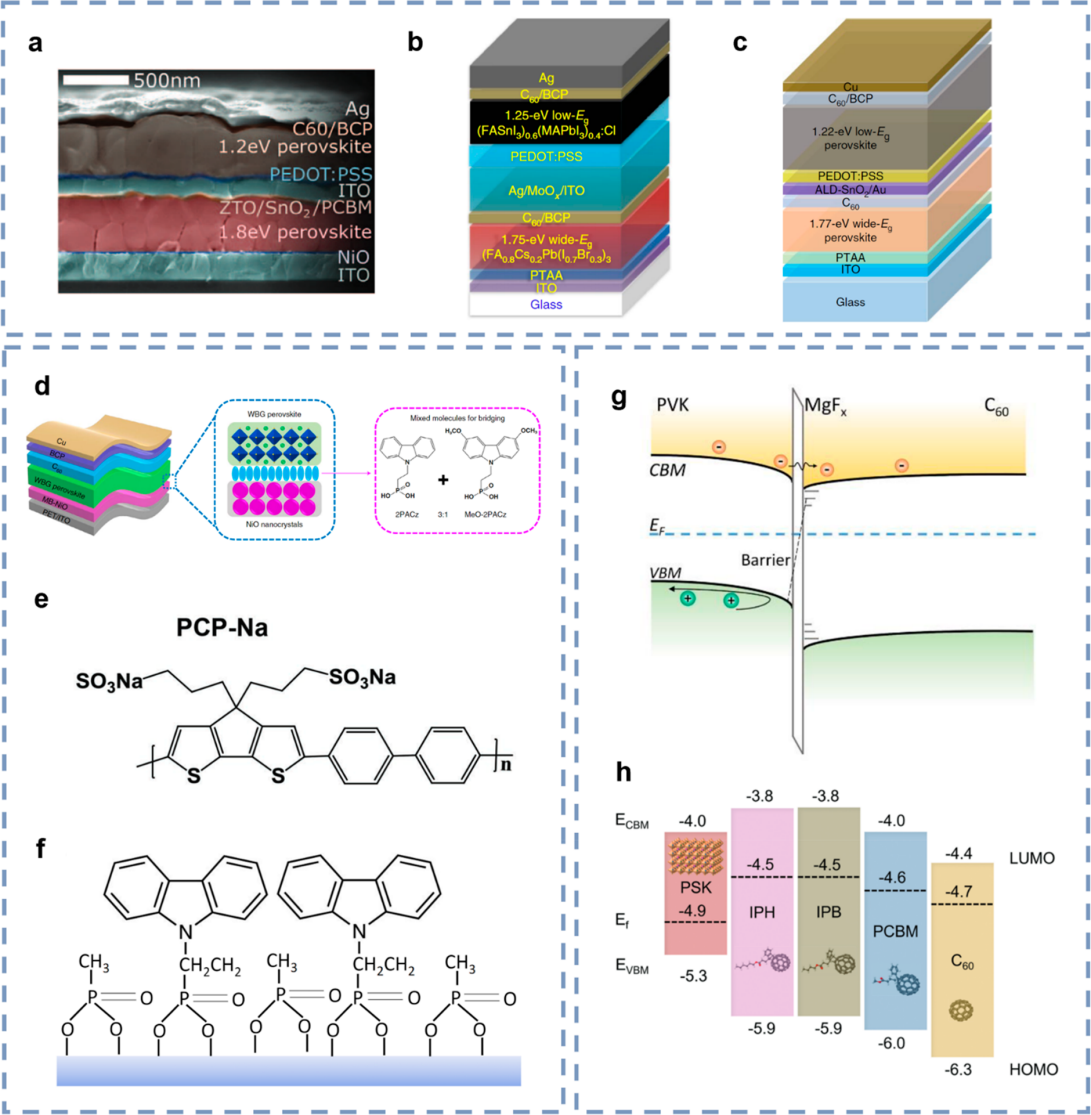
Optimization of functional layers for all-perovskite tandem solar cells. (a–c) Structure evolution of ICL in monolithic all-perovskite tandem solar cells. (d) Device and molecular structures of HTL. (e) Chemical structure of PCP-Na. (f) Schematic illustration of 2PACz/MPA bilayer formation on the substrate. (g) Energy level diagram of the perovskite/C60 interface with MgF_*x*_ insertion layer. (h) Energy level diagram of NBG perovskite and different fullerene derivatives. Panels (a) and (g) reproduced with permission from refs (69 and 74), respectively. Copyright 2016 and 2022 American Association for the Advancement of Science, respectively. Panels (b)–(d) reproduced with permission from refs (50, 70, and 75), respectively. Copyright 2019, 2018, and 2022 Springer Nature, respectively. Panels (e) and (h) reproduced with permission from refs (55 and 76), respectively. Copyright 2018 and 2022 Wiley, respectively. Panel (f) reproduced with permission from ref (77). Copyright 2022 American Chemical Society.

Previously, sputtered indium tin oxide (ITO) was widely used in ICL for protecting the underlying device layers from solvent damage during subsequent processing. For example, Snaith and co-workers used stacks of atomic layer deposition (ALD)-SnO_2_/(zinc-tin-oxide) ZTO/sputtered-ITO as the recombination layer for all-perovskite tandem devices (Figure 4a).^69^ Yan and co-workers reported a thermally evaporated multilayer structure of Ag(1 nm)/MoO_*x*_(3 nm)/sputtered-ITO(∼120 nm) in ICL (Figure 4b).^70^ However, the thick sputtered-ITO layer not only causes high-energy sputtering damage to the front subcell but also increases parasitic absorption and lateral shunting losses, resulting in an unfavorable effect on tandem devices.

It has been demonstrated that an ultrathin nucleation layer, consisting of poly(ethylenimine) ethoxylated (PEIE) with nucleophilic hydroxyl and amine functional groups, could be used prior to ALD layer deposition to facilitate the formation of a dense and conformal aluminum-doped ZnO (AZO) layer.^71^ The PEIE-nucleated AZO provides better protection against the solvent, enabling a thinner layer of sputtered-ITO (5–15 nm). Furthermore, our team successfully fabricated highly efficient all-perovskite tandem solar cells with the ICL configuration of C_60_/ALD-SnO_2_/Au/PEDOT:PSS (Figure 4c).^49,50,64^ This shows that the ALD-SnO_2_ layer is compact and robust enough to protect the underlying perovskite layer from damage during the solution processing of the rear subcell, without the necessity of sputtered-ITO. It is to be noted that an ultrathin Au layer deposited by thermal evaporation is essential to ensure efficient hole–electron recombination, without which the tandem devices exhibit a noticeable S-shape in the *J*–*V* curves.

Most recently, several efforts have been made to simplify the architecture of tandem photovoltaics for higher optical properties and better stability. Huang and co-workers developed a simplified ICL with only C_60_ and a SnO_2–*x*_ (0 < *x* < 1) layer, enabling tandem devices up to 24.4% and maintaining 94% of its initial performance after 1 000 h illumination.^72^ However, precise control of the oxidation state of Sn in SnO_*x*_ is a barrier to reproduce tandem solar cell performance. In a recent study, Riedl and co-workers developed an ultrathin ALD-indium oxide layer (∼1.5 nm) to connect the WBG perovskite and organic semiconductors, which showed better optical transmission and boosted the overall *J*_sc_ of the tandem device by ∼1.5 mA cm^–2^ compared with the traditional 1 nm Ag.^73^ This may also be favorable in all-perovskite tandem devices to enhance the matched current and reduce the risk of metal diffusion in long-term stability.

### 4.2 Charge-Transport Layers

In the architecture of tandem devices, the photogenerated charge carriers in each absorber are mainly extracted and transported by charge-transport layers on both sides, including the hole-transport layer (HTL) and the electron-transport layer (ETL). Suitable HTL and ETL can facilitate the charge carrier extraction and reduce undesired recombination, playing crucial roles in achieving high PCE and good stability of devices.

In a p-i-n device structure, the selection of an efficient HTL varies from organic polymeric to inorganic p-type semiconductors, such as PEDOT:PSS, poly[bis(4-phenyl)(2,4,6-trimethylphenyl)amine] (PTAA), nickel oxide (NiO_*x*_), and copper thiocyanate (CuSCN). For WBG perovskites, PTAA and NiO_*x*_ are commonly used as HTLs at the early stage, and recently self-assembled monolayers (SAMs) have emerged as a popular choice of HTL. The molecule of SAMs typically consists of an anchoring group that bonds to the surface of perovskites via chemical interaction, a terminal group that changes the surface or interface properties, and a linkage that bridges the anchoring and terminal groups.^78^ Al-Ashouri et al. found that Me-4PACz([4-(3,6-dimethyl-9*H*-carbazol-9-yl)butyl]phosphonic acid) provides fast hole extraction and good passivation at the interface.^79^ These combinational effects enable not only high performance of the perovskite/silicon tandem device (a certified PCE of 29.15%) but also promising stability with the unencapsulated tandem device (maintaining 95% of its initial PCE after 300 h of operation). Li et al. also reported a strategy of anchoring a mixture of SAMs on the NiO nanocrystals layer (Figure 4e), which assisted in matching energy alignment, facilitating hole extraction, and mitigating interfacial recombination for a ∼1.75 eV WBG perovskites, resulting in 16.2% PCE for a flexible single WBG perovskite and 24.7% for a flexible all-perovskite tandem device.^75^

On the other hand, PEDOT:PSS is the most widely used HTL in Pb–Sn perovskite cells, and these yield a high PCE of >23%.^62,64,65,68,80,81^ However, there is a growing concern about the adverse effects of PEDOT:PSS on the long-term stability of devices because of its acidic and hygroscopic nature. Loi and co-workers used PCP-Na, a neutral polymer (Figure 4f) used as an HTL for FAPb_0.5_Sn_0.5_I_3_ perovskites, which showed high performance.^55^ Recently, Hayase and co-workers employed [2-(9*H*-carbazol-9-yl)ethyl]phosphonic acid (2PACz) and methylphosphonic acid (MPA) as an HTL for Pb–Sn NBG perovskites and achieved a high PCE of 23.3% (Figure 4g).^77^ The utilization of SAMs in WBG or NBG perovskite single-junction cells has proven to be beneficial for both device efficiency and stability, and the further exploration of SAMs as an HTL in tandem devices is promising. Alternatively, NiO_*x*_, a metal oxide as an HTL, has been found to provide better performance and thermal stability compared to organic HTLs in Sn–Pb perovskite devices.^82^ However, surface defects and adverse chemical reactions at the NiO_*x*_/perovskite interface limit the performance of tandem devices.^83^ A better understanding of the NiO_*x*_/perovskite interface could improve performance, thereby leading to a wide adoption of NiO_*x*_ in tandem devices.^84^

HTL-free devices, that is, directly depositing perovskites on the ITO substrate, are also being explored.^85−88^ McGehee and co-workers reported that the perovskite band shifts upward near the interface with ITO, and this band offset facilitates hole extraction and effective electron blocking. HTL-free devices demonstrate excellent stability, retaining 95% of the initial efficiency after aging for 1 000 h at 85 °C in the dark without any encapsulation.^53^ However, the recombination rate and the matching in thermal expansion coefficients between perovskites and ITO layers all need to be further evaluated.

Unlike for HTLs, the choice of efficient ETLs in inverted devices is mainly limited to fullerenes and their derivatives, such as C_60_, IC_60_BA (indene-C60 bisadduct), and PC_61_BM ([6,6]-phenyl-C61-butyric acid methyl ester). The interface between perovskite and ETL has been demonstrated to be vital for the electron extraction and carrier recombination process. Warby and co-workers found that the C_60_ caused an undesignable energetic disorder upon the first contact with the Pb-based perovskite surface, leading to a substantial recombination loss, which is relatively independent of the perovskite composition.^89^ Liu and co-workers inserted an ultrathin fluoride interlayer, especially MgF_*x*_, between the perovskite and C_60_ interface to effectively mitigate C_60_-induced electronic disorders (Figure 4h). The perovskite/silicon tandem devices with MgF_*x*_ demonstrated a certified 29.4% efficiency and much-improved stability.^74^ Nejand and co-workers introduced indene-C60-propionic acid hexyl ester (IPH), a fullerene derivative, into the Pb–Sn perovskite/C_60_ interface, which delivered a spike-like structure in energy-level alignment and consequently suppressed nonradiative recombination (Figure 4i).^76^ Major work needs to be done in modifying ETLs to allow efficient carrier extraction and reduced nonradiative recombination at the perovskite/ETL interface. Furthermore, when choosing HTL/ETL for tandem devices, additional attention should be given to not only the ionization potential of the HTL/ETL but also the passivation/modification of interfaces to enhance the performance and stability of devices.

## 5 Conclusion and Outlook

**Figure 5:**
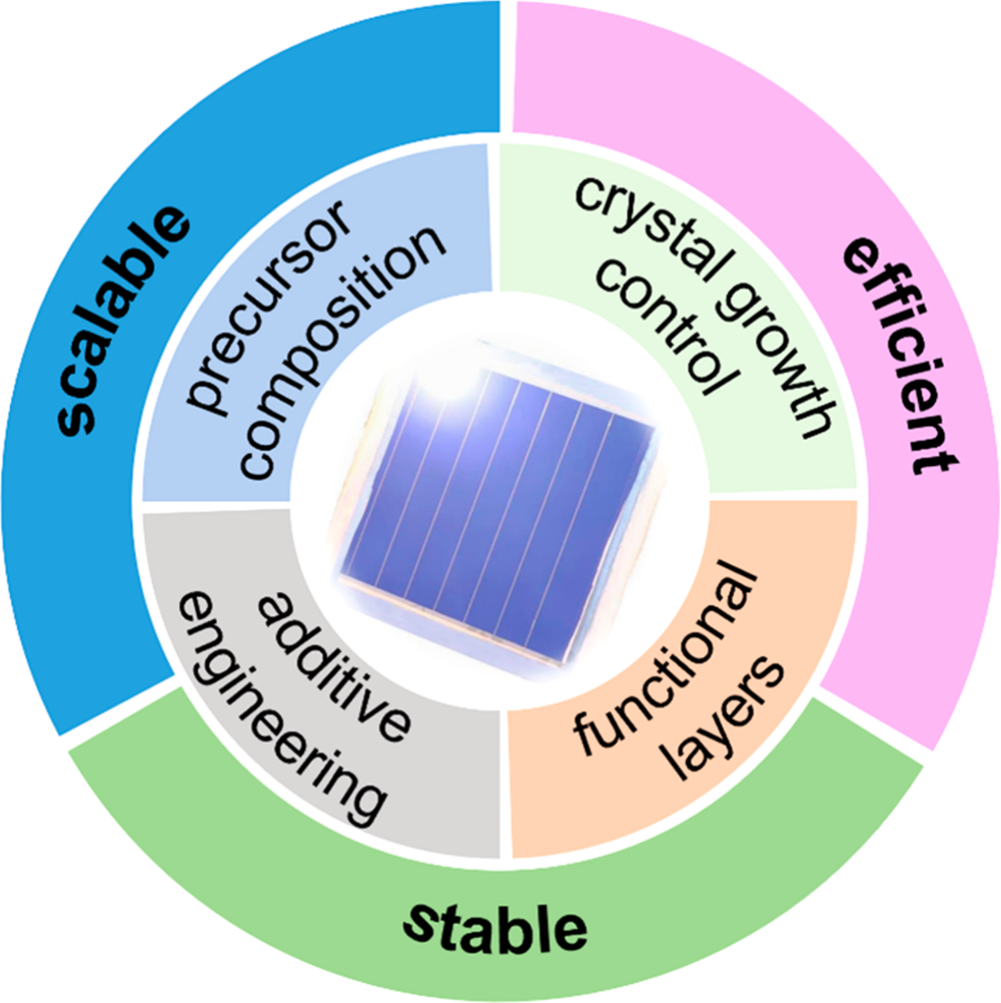
Outlook for all-perovskite tandem solar cells.

In summary, intensive research studies promote the enhancement of PCEs and stability for all-perovskite tandem solar cells, including perovskite composition management, crystal growth control, additive engineering, and functional layer optimization. Although the efficiency of monolithic all-perovskite tandem solar cells has reached 28.0%, greatly surpassing the record PCEs of single-junction photovoltaic devices, many challenges and opportunities remain. The efficient, scalable, and stable triangle can be used here to look toward the development of all-perovskite tandem solar cells in the near future (Figure 5).

### 5.1 Efficient

The voltage of tandem devices is limited by the WBG perovskite and offers room for improvement. Optimization of the bulk composition and interfaces, passivation of perovskite surface defects, and selection of charge-transport layers are promising approaches to enhance the performance and stability of the WBG subcell. For the NBG subcell, enhancing the thickness of the absorber layer has always been the key to getting higher matched current in all-perovskite tandem devices, and there is still a potential for enhancement according to the reported total absorptance curves.^64^ Additionally, innovative strategies to further reduce the perovskite bandgap are prospected to increase the current density by widening the absorption range. Moreover, reducing the parasitic absorption and reflection losses of functional layers, particularly in the long wavelength, before the NBG subcell is likely to increase the near-infrared response of the rear subcell and associated benefits on tandem devices.

### 5.2 Scalable

Large-scale manufacturing is an essential step in the commercialization of all-perovskite tandem devices. However, certified higher efficiencies were mainly achieved in lab-scale small-area devices, limiting scalability. Challenges in the large-scale fabrication of tandem devices include obtaining perovskite subcells with good homogeneity and developing scalable deposition strategies for functional layers. Typically, the perovskite precursor ink, which involves perovskite-composition management, additives engineering, and solvent selection, plays a critical role in the scalable deposition process. The differences in the perovskite precursor ink can modulate the crystallization processing, thus controlling the grain size, morphology, and crystallinity of the resultant films.^90^ Meanwhile, solvent quenching methods are also important to achieving pinhole-free and uniform perovskite layers, which should be coupled with perovskite precursor characteristics. Our team reported that the crystal orientation and crystallinity of a WBG perovskite could be precisely controlled by tuning the Cs content when deposited by a blade-coating method. An appropriate precursor ink in conjunction with a gas quenching technique resulted in all-perovskite tandem modules (aperture area of 20.25 cm^2^) with a certified PCE of 21.7%.^91^ Additionally, further research on optimizing the interconnection structures, improving the air-resistant ability of perovskite absorbers (particularly for NBG perovskite materials), and developing green solvents is urgently needed to advance the practical application of all-perovskite tandem devices.

### 5.3 Stable

Regardless of the impressive progress on PCE enhancement, commercialization of all-perovskite tandem devices is limited by the intrinsic and extrinsic instability of perovskites. It is of vital importance for researchers to reveal the exact degradation mechanisms both within the perovskite absorbers and at the interface between perovskite/functional layers via advanced techniques, such as in situ, nanoscale, or less-destructive characterizations. Then, targeted strategies could be developed to synergistically improve the photostability, thermal stability, and humid stability for perovskite solar cells. It is also encouraged to perform more aggressive stability tests, like outdoor testing and thermal cycling testing, for better estimating the real lifetime of perovskite solar cells. Moreover, the design of novel tandem architectures and encapsulation technology is an effective and practical way to improve the long-term stability of perovskite solar cells under ambient conditions, for example, replacing the metal electrode with a transparent conductive oxide electrode or developing a low-temperature packing technique.
